# DSG2 promotes pancreatic cancer stem cell maintenance via support of tumour and macrophage cellular cross-talk

**DOI:** 10.1038/s41419-025-07833-4

**Published:** 2025-07-04

**Authors:** Faming Wang, Tao Sun, Ning Wang, Wei Wei, Ying Mei, Qiang Yan

**Affiliations:** 1 The Key Laboratory of Molecular Medicine, Huzhou Central Hospital, Fifth School of Clinical Medicine of Zhejiang Chinese Medical University, Huzhou, Zhejiang China; 2 Huzhou Central Hospital, Affiliated Central Hospital of Huzhou University, Huzhou, Zhejiang China; 3 Clinical Laboratory, The Key Laboratory of Molecular Medicine, Huzhou Central Hospital, Huzhou, Zhejiang PR China; 4 Department of Hepatobiliary and Pancreatic Surgery, Fifth School of Clinical Medicine of Zhejiang Chinese Medical University, Huzhou Central Hospital, Huzhou, China; 5 Department of Surgery, Affiliated Huzhou Hospital, Zhejiang University School of Medicine, Huzhou, China; 6 Department of Surgery, Affiliated Central Hospital of Huzhou University, Huzhou, China; 7 Huzhou Key Laboratory of intelligent and digital precision Surgery, Huzhou, China

**Keywords:** Oncogenes, Cancer stem cells

## Abstract

Pancreatic cancer stem cells (PCSCs) are a small population of cells in tumours that exhibit enhanced self-renewal and differentiation capabilities. CSCs proactively remodel the tumour microenvironment to maintain CSC stemness, which contributes to chemotherapy resistance. Compared with targeting PCSCs themselves, targeting the PCSC niche may be a novel strategy for pancreatic cancer (PC) therapy. Here, we found that DSG2, a member of the desmosomal cadherin family, is highly expressed in PCSCs. DSG2 upregulation is correlated with adverse outcomes in PC patients. DSG2 knockdown suppressed IL-4 and GM-CSF expression, which promoted the enrichment of tumour-associated macrophages to establish a supportive PCSC niche. Furthermore, we found that the IL-8/CXCR2 axis interacts with DSG2 to promote PCSC stemness and gemcitabine resistance by activating the Wnt/β-catenin pathway. These findings highlight the novel regulatory mechanism of DSG2 in PC, providing new targets for the development of therapeutics targeting PCSC niches.

## Introduction

Pancreatic cancer (PC) is a prevalent malignant gastrointestinal tumour known for its high potential for metastasis and the difficulty of detecting the disease. Notably, the 5-year survival rate after surgery is only 10% [1]. One key characteristic of PC is its notable resistance to most conventional therapies, including gemcitabine [2]. Recently, the literature has revealed the significant role of cancer stem cells (CSCs) in tumour resistance [3]. CSCs possess quintessential stem cell traits, such as self-renewal, tumour initiation and tumour sustenance abilities. They typically differentiate into diverse cell populations to varying extents, present metastatic and invasive tendencies, and exhibit resistance to anticancer medications and radiation [4]. CSCs are recognised in multiple cancer types, including lung, breast, liver, colon and prostate cancers [5]. In the context of PC, CD133, CD44 and EpCAM have been identified as pancreatic cancer stem cell (PCSC) markers [6]. Tumorigenicity assays revealed that CD44^+^EpCAM^+^ PC cells demonstrated enhanced tumorigenicity in mice [7]. Similarly, CD133^+^ PC cells exhibit increased tumorigenicity [8]. Hence, targeting PCSCs is a highly promising strategy for PC treatment. However, evidence indicates that CSCs proactively remodel the tumour microenvironment (TME) to maintain CSC stemness, which contributes to chemotherapy resistance [9]. Therefore, targeting the PCSC niche may be a novel strategy for PC therapy compared with targeting PCSCs themselves.

Desmoglein-2 (DSG2), located on chromosome 18q12.1, is a member of the desmosomal cadherin family, which not only participates in intercellular connectivity and desmosome assembly but also plays an important role in the migration and invasion of cancer cells [10]. Numerous studies have shown that DSG2 is overexpressed in various tumours, including cervical, anaplastic thyroid, colon and gastric cancers. Consequently, DSG2 has been recognised as a prognostic marker in solid tumours [11]. Moreover, DSG2 can promote the stemness of CSCs [12]. High expression of DSG2 increased tumour resistance to osimertinib in lung cancer [13]. However, the role of DSG2 in PCSCs and the underlying mechanism have been poorly reported, especially with respect to the cross-talk of the PCSC niche, which remains unclear.

In this study, we discovered that DSG2 is highly expressed in PCSCs and is correlated with a poor prognosis. DSG2 knockdown (KD) impaired the expression of IL-4 and GM-CSF, which both mediate cross-talk between cancer cells and stromal cells in the PCSC niche. Furthermore, we demonstrated that the IL-8/CXCR2 axis interacts with DSG2, promoting the stemness and gemcitabine resistance of PCSCs through the Wnt/β-catenin pathway. Therefore, our innovative findings suggest that DSG2 is crucial for PC tumorigenesis and the maintenance of stemness, which provides a basis for further research on DSG2 as a potential target for cancer treatment strategies.

## Materials and methods

### Cell culture and transfection

PC cell lines (PANC-1, MIAPaCa-2), 293T cells and THP-1 cells were acquired from the American Type Culture Collection (Manassas, VA, USA). PANC-1, MIAPaCa-2 and 293T cells were cultivated in DMEM (catalogue no. 209011; NEST Biotechnology). THP-1 cells were grown in RPMI 1640 medium (catalogue no. 209021; NEST Biotechnology). The medium was supplemented with 10% heat-inactivated foetal bovine serum (catalogue no. abs972; Absin, Shanghai, China) and penicillin‒streptomycin solution (Beijing Solarbio Science & Technology Co., Ltd.). All the cultures were maintained at 37 °C in a humidified atmosphere containing 5% CO2 in an incubator (Thermo Fisher Scientific). Sh-DSG2#1, #2 and #3 were generously provided by TranSheepBio (Shanghai, China). Nontargeted control shRNA (sh-Control) was obtained from Addgene (pLKO.1 TRC control, catalogue no. 10879).

### Development of Gemcitabine-Resistant PDAC Cell Lines

Gemcitabine-resistant pancreatic ductal adenocarcinoma (PDAC) cells were developed in the MIAPaCa-2 and PANC-1 cell lines through prolonged exposure to escalating doses of gemcitabine (MCE, Guangzhou, China) for ~3 months. Specifically, the parental cell lines were subjected to gemcitabine concentrations ranging from 1 nM to 10 μM for 72 h, and cell viability was assessed using the Cell Counting Kit-8 (CCK-8; Dojindo Laboratories, Kumamoto, Japan). Subsequently, MIAPaCa-2 and PANC-1 cells were treated with gemcitabine at an initial concentration below their respective IC50 values. Once the cells adapted to this concentration, the gemcitabine dose was incrementally increased to 1 μM. This iterative process led to the establishment of gemcitabine-resistant cell lines, which were named MIAPaCa-2/Gem and PANC-1/Gem. The resistance index (RI) was calculated by dividing the IC50 of the resistant cells by the IC50 of the parental cells.

### Mouse xenograft models

Healthy 6-week-old nude mice and C57BL/6 mice were obtained from the Comparative Medicine Center of Yangzhou University (*n* = 50/group). They were housed under standardised laboratory conditions. All experimental animals were maintained in a specific-pathogen-free (SPF) facility. The animal study was approved by the Ethics Committee of Huzhou Central Hospital (202403001, dated March 5, 2024). Tumour growth was monitored every 3 days, and the volume was calculated via the following formula: volume = (length × width²)/2. Tumour tissue samples were acquired and subjected to trypan blue staining. The animal experiments were carried out in accordance with procedures established in our previous study [14].

### Macrophage Depletion

Clodronate liposomes (200 μl) were administered via intraperitoneal injection on days 1, 8, 15 and 22. Clodronate liposomes were obtained from Liposomes (CP-005-005) PBS were used as a control. On day 28, the tumours from the mice were isolated for analysis.

### Immunohistochemistry

The tumour samples were processed and sectioned as described previously [15]. The tissue sections were then blocked with protein blocks and incubated overnight at 4 °C with antibodies against DSG2, CD68, CD206, CD133 and Ki-67. Immunodetection was performed the following day using a 3,3’-diaminobenzidine (DAB) kit according to the manufacturer’s instructions, and the samples were counted after haematoxylin staining. The images were analysed via ImageJ software (http://rsb.info.nih.gov/ij/).

### Transwell assay

THP-1 cells were incubated with PMA (50 nM) at 37 °C for 3 h to induce differentiation into macrophages, and then migration assays were performed. The migration assays were performed using a Transwell chamber (Corning, NY, USA). THP-1 cells (1 × 10^5^) in 400 µl of serum-free medium were placed into the upper chamber, and PANC-1 cells were placed in the lower chamber. After being incubated for 8 h, the cells on the upper membrane were fixed with 4% paraformaldehyde (PFA) and stained with 0.2% crystal violet (Sigma). The cells on the upper membrane were removed with cotton swabs. The stained cells were counted under a microscope in three independent 200× fields for each well.

### Colony formation assay

After being washed with PBS, the cells were resuspended in fresh total DMEM. Transfected cells were plated into a 6-well plate at a density of 1000 cells per well. For the soft agar colony formation assay, the cells were mixed with agarose in growth medium at a final concentration of 0.4% agarose. The cell mixture was plated on top of a solidified layer of 0.8% agarose in growth medium. The cells were fed every 7 days with growth medium containing 0.4% agarose. The cells were incubated at 37 °C with 5% CO2 for 2 weeks. The colonies were fixed in 4% PFA (Solarbio, China) for 30 min and stained with crystal violet (Solarbio, China).

### Quantitative real-time PCR (qRT‒PCR)

Total RNA was extracted from cells using TRIzol reagent (Beijing Solarbio Science & Technology Co., Ltd.). In accordance with the manufacturer’s instructions, 1 μg of RNA was converted to cDNA using the HiScript II 1st Strand cDNA Synthesis Kit (Cat No. 11119-11141; Yeasen, Shanghai, China). Real-time quantitative PCR was performed using SYBR Green Pro Taq HS (Cat No. AG11701; Accurate Biotechnology Co., Ltd., Changsha, China). The relative expression of target genes was calculated via the 2^−ΔΔCT^ method. The sequences of the primers used in this research are listed in Supplementary Table 1.

### Western blotting (WB)

The proteins were isolated as previously described [16]. For membrane protein extraction, the cells were collected at 4 °C and washed twice with precooled PBS. The membrane protein was extracted using a membrane protein extraction kit (Proteintech, USA) following the manufacturer’s instructions. The protein concentration was determined using the BCA Protein Assay Kit (Beijing Solarbio Science & Technology Co., Ltd.). After separation via SDS‒PAGE, the proteins were transferred onto PVDF membranes (Shanghai Acmec Biochemical Co., Ltd. Shanghai, China) and incubated with the corresponding primary antibodies at 4 °C overnight. The protein bands were quantified using a Gel-Pro Analyzer (Media Cybernetics, Rockville, MD, USA). The primary antibodies and subset definitions used in this research are listed in Supplementary Table 2.

### ELISA assays

Cell culture supernatant was collected and centrifuged at 1000 × *g* for 30 min at 4 °C. The clarified supernatant was used for analysis. Standard curves were established by adding 50 µl of varying concentrations of pre-diluted standards to designated wells. Subsequently, 50 µl of each test sample was added to the respective wells. Next, 50 µl of horseradish peroxidase (HRP) conjugate was added to each well and incubated for 1 h at 37 °C. After incubation, the liquid was removed and the wells were washed with 300 µl of wash buffer. The plate was allowed to sit for 2 min before discarding the wash buffer and blotting the plate dry on absorbent paper to remove any residual liquid. The washing process was repeated five times to ensure thorough cleaning. Finally, 50 µl of substrate solution was added to each well and incubated for 15 min. The reaction was stopped by adding 50 µl of stop solution, and the absorbance of each well was measured at 450 nm.

### Sphere culture assay

After being washed with PBS, the cells were seeded in ultralow attachment plates in serum-free DMEM containing B27 (Invitrogen), 20 ng/ml bFGF (Invitrogen), 10 ng/ml EGF (Invitrogen) and 5 μg/ml insulin (Invitrogen). The medium was replaced with fresh stem cell medium twice a week. After 2–3 weeks of cultivation, the number of tumour spheres formed in each well was quantified.

### Flow cytometry

The cells were washed twice with staining buffer (PBS, 1% FBS), and apoptosis status state was detected with an Annexin V-FITC apoptosis detection kit (Vazyme). All samples were incubated with Annexin V-FITC or PI dye and filtered immediately before analysis to remove any clumps. For cell surface marker analysis, the cells were stained with anti-CD133-FITC (BD, 1:400) for 30 min at 4 °C. Flow cytometry was performed on an LSRII, and the data were analysed via FlowJo software. The results were examined via ModFit-LT software.

### Co-immunoprecipitation (co-IP) assays

Protein lysates were prepared using NP-40 buffer (Beyotime Biotechnology, P0013F) and measured via BCA method. The lysates were first incubated with primary antibodies against human CXCR2 (Proteintech, 20634-1-AP; 1:100) at 4 °C overnight. Subsequently, Protein G Magnetic Beads (MCE, HY-K0204) were added and the mixture was incubated for an additional 4 h. The beads were then washed thoroughly with PBS for five times and the proteins were eluted using elution buffer. The co-immunoprecipitated complexes were ultimately analysed by immunoblotting.

### Bioinformatics analysis

Gene expression profiling interactive analysis (GEPIA) was subsequently performed (http://gepia.cancer-pku.cn/index.html). The RNA sequencing expression data for the PCSCs were obtained from previous studies [17–19]. The original expression matrix files were downloaded from the NCBI Gene Expression Omnibus (GEO) database. Gene sets with a nominal *p* < 0.05 and a false discovery rate (FDR) value ≤25% were considered significantly enriched.

### Statistical analysis

All the statistical analyses were performed via SPSS 23.0 (IBM Corp., Armonk, NY, USA) or GraphPad Prism 9.0 software (San Diego, CA, USA). The difference in the expression of each molecule in the ranked data was calculated via the chi-square test. Survival curves were plotted on the basis of the follow-up data via the Kaplan–Meier method, and cumulative survival rates in different groups were compared via the log-rank test. The data were obtained from at least three independent experiments. For all analyses, a statistically significant difference is denoted as *p* < 0.05* and *p* < 0.01**.

## Results

### Identification of differentially expressed genes (DEGs) between PCSCs and control cells

**Fig. 1:**
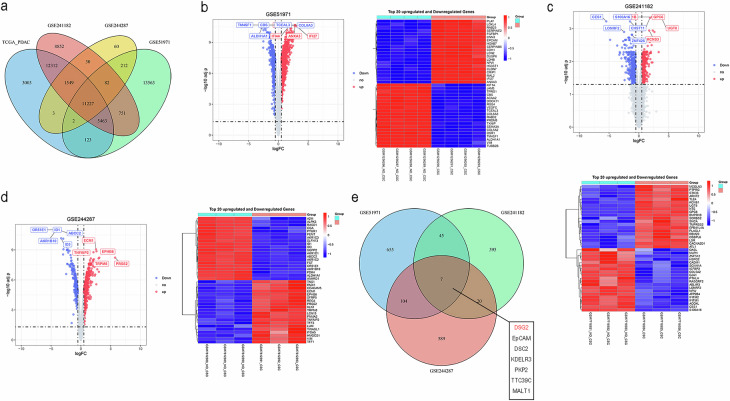
Identification of differentially expressed genes (DEGs) in pancreatic cancer stem cells (PCSCs). **a** A Venn diagram depicting the overlap of expressed genes in the Gene Expression Omnibus (GEO) and The Cancer Genome Atlas (TCGA) datasets. **b** Volcano plot and heatmap showing the distribution of DEGs in GSE51971. The blue colour represents downregulated genes, and the red colour represents upregulated genes. **c** Volcano plot and heatmap showing the distribution of DEGs in GSE241182. The blue colour represents downregulated genes, and the red colour represents upregulated genes. **d** Volcano plot and heatmap showing the distribution of DEGs in GSE244287. **e** Venn diagram of the overlapping upregulated genes in the GEO datasets (GSE51971, GSE241182 and GSE244287). PCSCs pancreatic cancer stem cells, TCGA The Cancer Genome Atlas.

To identify critical genes involved in PCSC progression, we explored existing published RNA-seq data from the GEO and The Cancer Genome Atlas (TCGA) databases. In the related studies, PCSCs were collected mainly through magnetic bead separation (CD133^+^CD44^+^EpCAM^+^) and sphere enrichment under suspension culture conditions [20]. A total of 11,227 PCSCs were identified via analysis of RNA sequencing and clinical data (Fig. 1a). The volcano plot shows 811 upregulated genes identified in the GSE51971 dataset. The heatmap displays the top 20 genes with the most significant upregulation and downregulation (Fig. 1b). Similarly, 467 and 720 upregulated genes were obtained from the GSE241182 dataset and the GEO244287 dataset, respectively (Fig. 1c, d). A list of common and specific DEGs is shown in Supplementary Table 1. Finally, a total of 7 overlapping highly expressed genes were identified in the above three datasets (Fig. 1e). To identify critical genes involved in PCSC progression, we conducted survival analyses. We found that 2 genes, DSG2 and DSC2, had detrimental influences on patient OS (Supplementary Fig. S1). DSC2 has been well characterised in pancreatic ductal adenocarcinoma (PDAC) [21]; therefore, we focused this study on DSG2 in PCSCs.

### DSG2 expression predicted poor tumour prognosis and immune cell infiltration across cancers

**Fig. 2:**
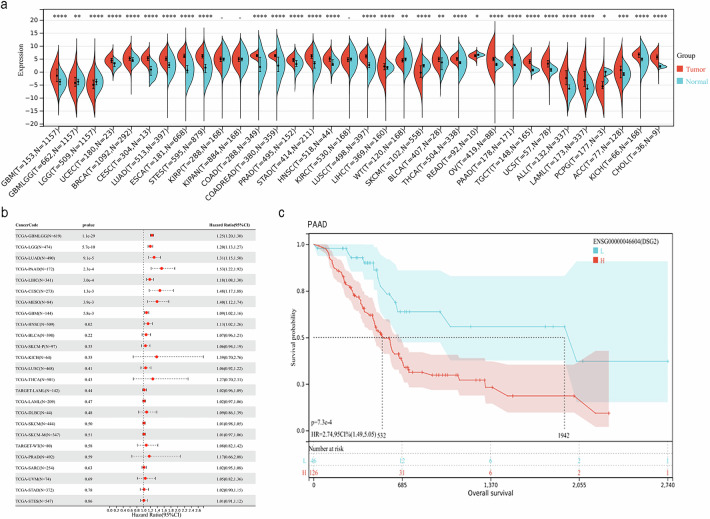
DSG2 upregulation predicted poor tumour prognosis and immune cell infiltration across cancers. **a** Differential expression of DSG2 in various tumour tissues and adjacent normal tissues. **b** Forest plot of overall survival in different tumour types. **c** Impact of high DSG2 expression on the survival probability of patients with PDAC.

To further clarify the potential prognostic value of DSG2 in cancer, we investigated its expression across cancers via the Sangerbox website. Analysis of data from the TCGA revealed that DSG2 was upregulated in 25 of 34 tumour types (Fig. 2a). These analyses collectively demonstrate that DSG2 is significantly upregulated in multiple carcinoma types and has the potential to serve as a cancer biomarker. Moreover, the OS rates of 25 DSG2-overexpressing cancers were evaluated (Fig. 2b). Kaplan‒Meier survival curves were generated according to the following cancer datasets: glioma (GBMLGG), brain lower grade glioma, lung adenocarcinoma, pancreatic adenocarcinoma, liver hepatocellular carcinoma, cervical squamous cell carcinoma and endocervical adenocarcinoma, mesothelioma and glioblastoma multiforme (Supplementary Fig. S2a–g). These results suggest that DSG2 upregulation has a detrimental effect on patient OS, especially in patients with PDAC. DSG2 is a member of the desmosomal cadherin family, which may play a role in tumour–stroma crosstalk [22]. To explore the potential association between DSG2 expression and CSC niche features, we analysed the distribution of immune cells through different algorithms (Supplementary Fig. S2h). Macrophages were more abundant than other immune cells, suggesting their critical role in the DSG2-mediated CSC niche.

### DSG2 enhances the stemness of PCSCs

**Fig. 3:**
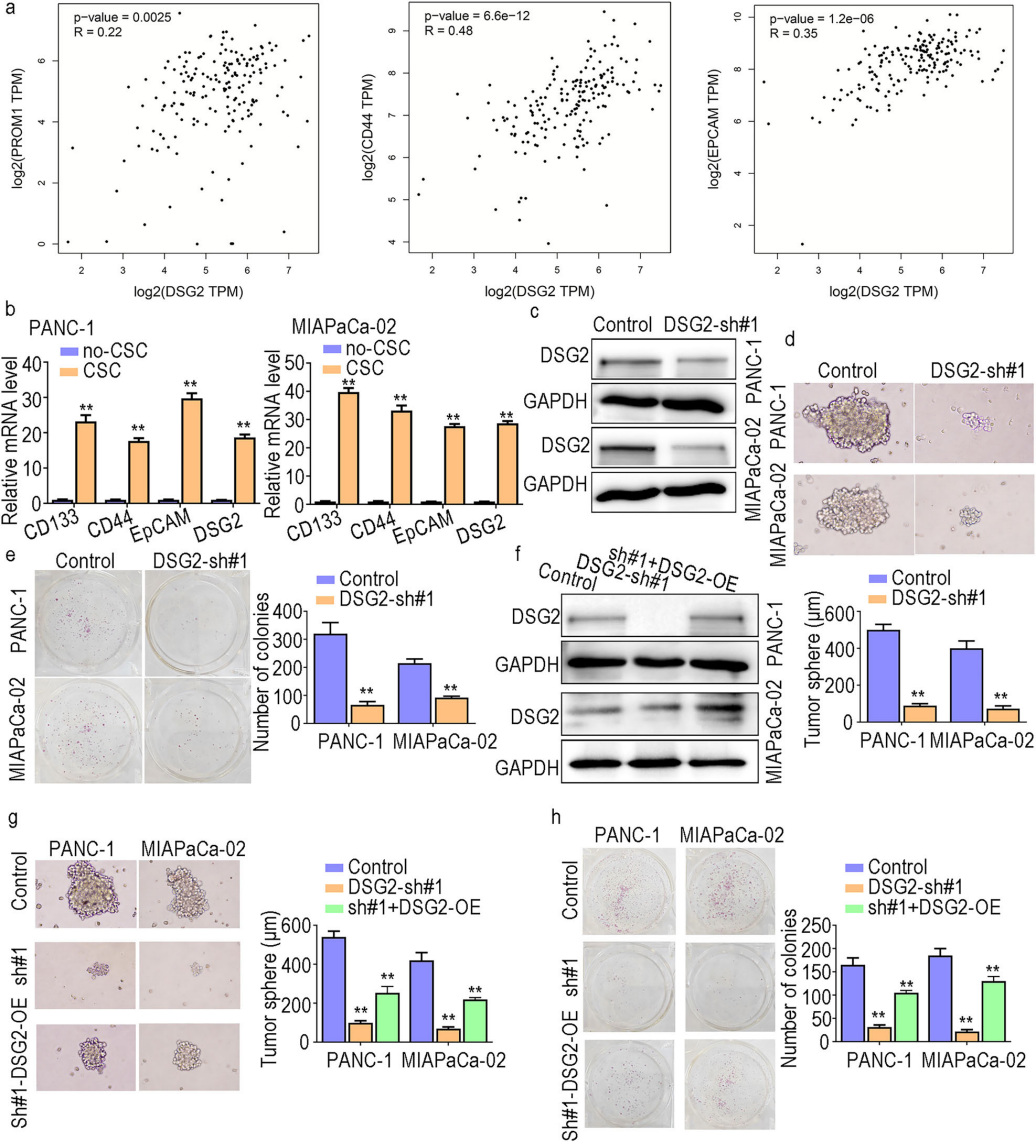
DSG2 knockdown inhibits the stemness of PCSCs in vitro. **a** Expression correlations between DSG2 and CD133, CD44, or EpCAM were determined via bioinformatics analysis. **b** The expression level of DSG2 in CSCs and non-CSCs isolated from populations of PANC-1 and MIAPaCa-02 cells was analysed via RT‒PCR. **c** Construction of DSG2-downregulated cell lines. **d** A sphere formation assay was performed to compare the control and sh-DSG2 groups of PANC-1 and MIAPaCa-02 cells on day 7. **e** Colony formation assays were carried out to validate the role of DSG2 in the proliferation of PANC-1 and MIAPaCa-02 cells. **f** WB assays were performed to assess the upregulation efficiency of DSG2. **g**, **h** Overexpression of DSG2 restored cell stemness via sh-DSG2 inhibition. The data are presented as the mean ± standard deviation (SD) from three independent experiments. *P < 0.05, **P < 0.01.

**Fig. 4:**
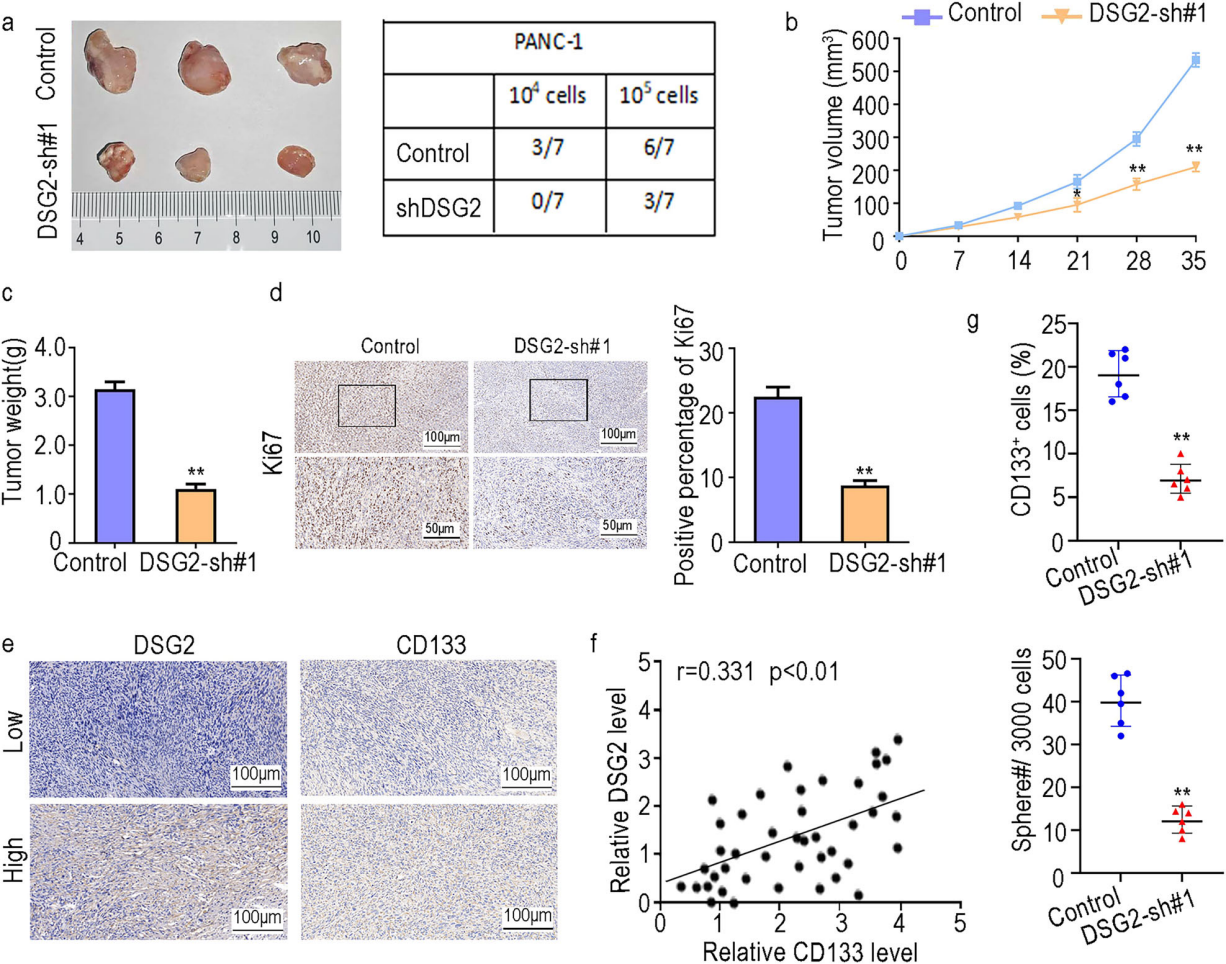
DSG2 knockdown inhibits the stemness of PCSCs in vivo. **a** Representative images of xenograft tumours in nude mouse. PANC-1 and MIAPaCa-02 cells were subcutaneously transplanted into nude mouse (10^5^ cells and 10^4^ cells per site, respectively). *n* = 7. **b**, **c** Tumour growth curves were generated to monitor the progression of tumours. **d** The expression level of Ki67 in tumours was analysed via immunohistochemistry (IHC). **e** IHC was performed to evaluate CD133 and DSG2 staining in fixed sections. **f** Trends in the expression levels of CD133 and DSG2 in tumours. **g** Xenograft tumorigenesis was analysed to detect the PCSC population and tumoursphere formation. The data are presented as the mean ± standard deviation (SD) from three independent experiments. *P < 0.05, **P < 0.01.

CD133, CD44 and EpCAM are markers of PCSCs [23]. PDAC patients with high CD133, CD44 or EpCAM expression generally have a poor prognosis [24]. Thus, we analysed the expression levels of DSG2, CD133, CD44 and EpCAM via the GEPIA website and assessed the correlations among them. The results indicated that CD133, CD44 and EpCAM were strongly correlated with DSG2 expression in patients with PC (Fig. 3a). Similarly, the RT‒PCR results revealed that DSG2 expression was significantly elevated in PCSCs (Fig. 3b). These data further highlight the close association between DSG2 and the PCSC niche. We subsequently evaluated the functions of DSG2 in PCSC stemness via in vitro and in vivo assays. We established stable DSG2 gene knockdown cell lines via lentiviral transduction. The WB results indicated that DSG2 expression levels in the sh-DSG2#1 group were significantly lower than those in the control group (Fig. 3c). To elucidate the effects of DSG2 downregulation on the development of single cells into PCSCs, we conducted a sphere formation assay and a PCSC population detection assay. DSG2 silencing resulted in a substantial inhibitory effect on PCSC stemness (Fig. 3d; Supplementary Fig. S3a, b). Furthermore, we observed a marked reduction in the colony formation ability of PANC-1 and MIAPaCa-2 cells in the absence of DSG2 (Fig. 3e; Supplementary Fig. S3c). In addition, we overexpressed DSG2 in PANC-1 and MIAPaCa-2 cells via lentiviral transduction (Fig. 3f). The inhibition of PCSC enrichment and colony formation ability was reversed by DSG2 overexpression (Fig. 3g, h; Supplementary Fig. S3d). These results further confirmed the supporting role of DSG2 in PCSC stemness. Concurrently, we established a tumour xenograft nude mouse model to further confirm the role of DSG2 in vivo. Our results revealed significantly lower tumour-initiating capacity in the DSG2 knockdown group than in the control group (Fig. 4a). The growth rate of the tumours was also reduced (Fig. 4b, c), and the proportion of Ki67-positive cells was significantly decreased (Fig. 4d). The IHC results suggested that CD133 and DSG2 have similar expression trends in PDAC (Fig. 4e, f). Additionally, we detected PCSC stemness in the above xenograft tumours and found that the tumoursphere formation ability and CD133^+^ PCSC proportions decreased markedly in the knockdown group (Fig. 4g). Therefore, our findings emphasise that DSG2 plays a crucial role in tumour growth and stemness both in vitro and in vivo.

### DSG2 promotes cross-talk between PCSCs and M2 macrophages

**Fig. 5:**
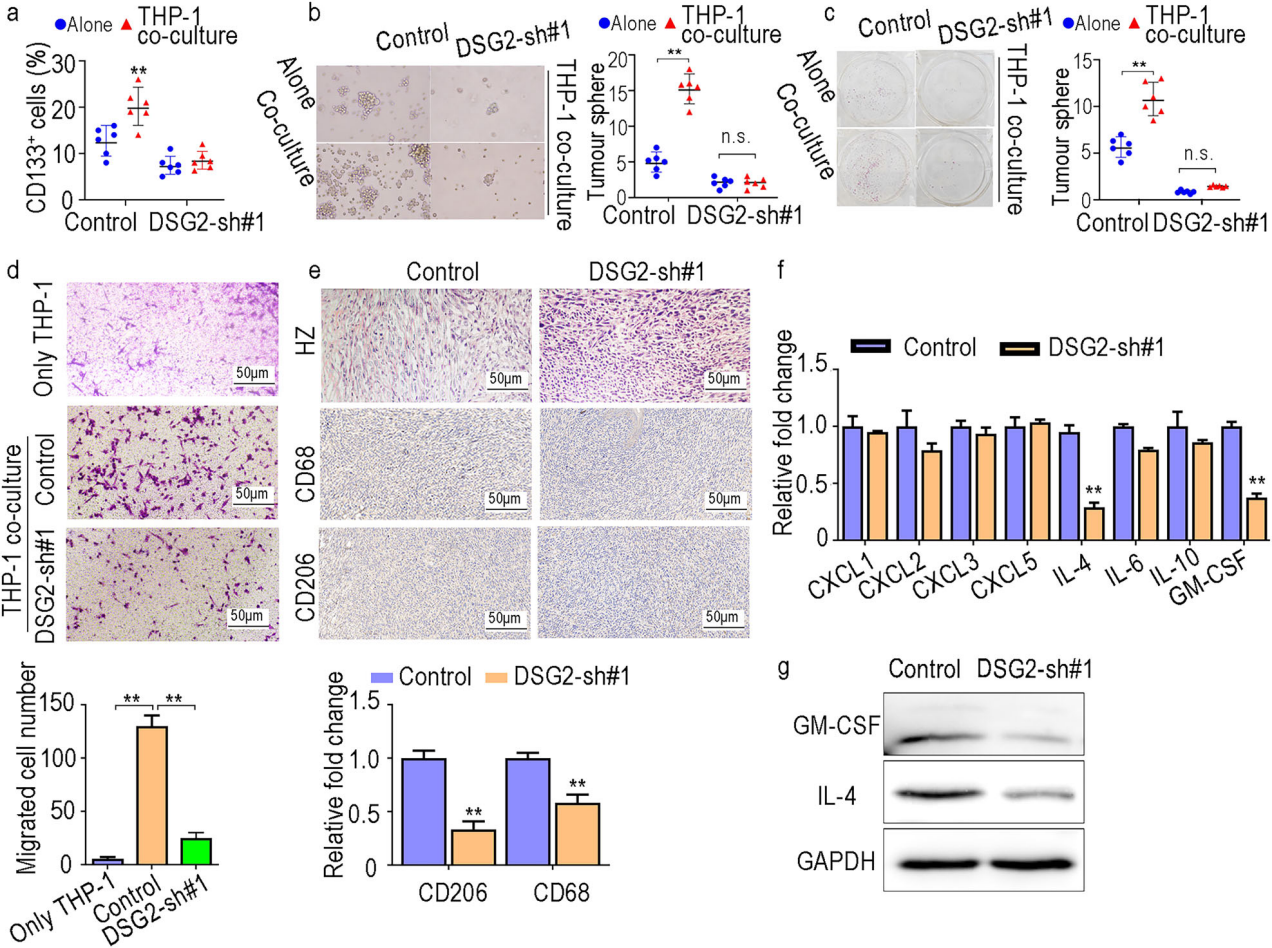
DSG2 promotes cross-talk between PCSCs and M2 macrophages. **a** The percentage of CD133^+^ cells among all cells was detected via flow cytometry. **b**, **c** Tumoursphere and colony formation assays in PANC-1 cells cocultured with PMA-primed THP-1 macrophages. **d** Transwell assays were used to assess the migration of macrophages in the coculture system. **e** The expression levels of CD68 and CD206 in tumour xenografts were determined via IHC staining. **f**, **g** Expression levels of cytokine genes. The data are presented as the mean ± SD from three independent experiments. *P < 0.05, **P < 0.01.

An important factor for the formation and maintenance of CSCs is the complex local TME, which includes tumour-associated macrophages [25]. Our previous results suggested that DSG2 may play a critical role in the macrophage-mediated CSC niche (Supplementary Fig. S2). To clarify the influence of DSG2 on the macrophage-mediated CSC niche, we performed a coculture assay. The FACS results revealed that DSG2 knockdown notably inhibited CD133^+^ cell enrichment in the coculture group (Fig. 5a). We also performed a tumoursphere formation assay and a colony formation assay under the above coculture conditions. Similarly, macrophages did not promote the stemness of PCSCs in the DSG2 knockdown groups (Fig. 5b, c). These results suggest that DSG2 plays a critical role in the progression of macrophages to support PCSC stemness. In addition, Transwell assays with macrophages revealed visibly reduced cell migration potential following DSG2 knockdown (Fig. 5d), indicating that DSG2 is involved in the recruitment of macrophages. Consequently, the results of the IHC staining of xenograft tumours further confirmed that the number of macrophages, especially the number of CD68^+^ macrophages (M2), significantly decreased in the DSG2-knockdown group (Fig. 5e; Supplementary Fig. S4a). To further elucidate the critical role of macrophages in DSG2-regulated PC progression, a mouse tumour model was established by subcutaneously implanting PANC02 cells that overexpress DSG2. The study found that overexpression of DSG2 significantly promoted tumour growth (Supplementary Fig. S4b). Moreover, depletion of macrophages using clodronate liposomes (CLD-lipos) markedly inhibited the growth of subcutaneous tumours in mice (Supplementary Fig. S4c). In tumorous tissue, cancer cells induce macrophage polarisation towards the M2 phenotype with different cytokines, which establishes an inhibitory niche [26]. Therefore, we further investigated the expression of cytokine genes, including CXCL1, CXCL2, CXCL3, CXCL5, IL-4, IL-6, IL-10 and GM-CSF. The RT‒PCR, and WB, and ELISA results revealed a pronounced decrease in the expression levels of IL-4 and GM-CSF (Fig. 5f, g; Supplementary Fig. S4d). Taken together, these data suggest that DSG2 promotes the recruitment of macrophages through GM-CSF and IL-10.

### DSG2 promotes macrophage-mediated PCSC stemness by directly binding to CXCR2

**Fig. 6:**
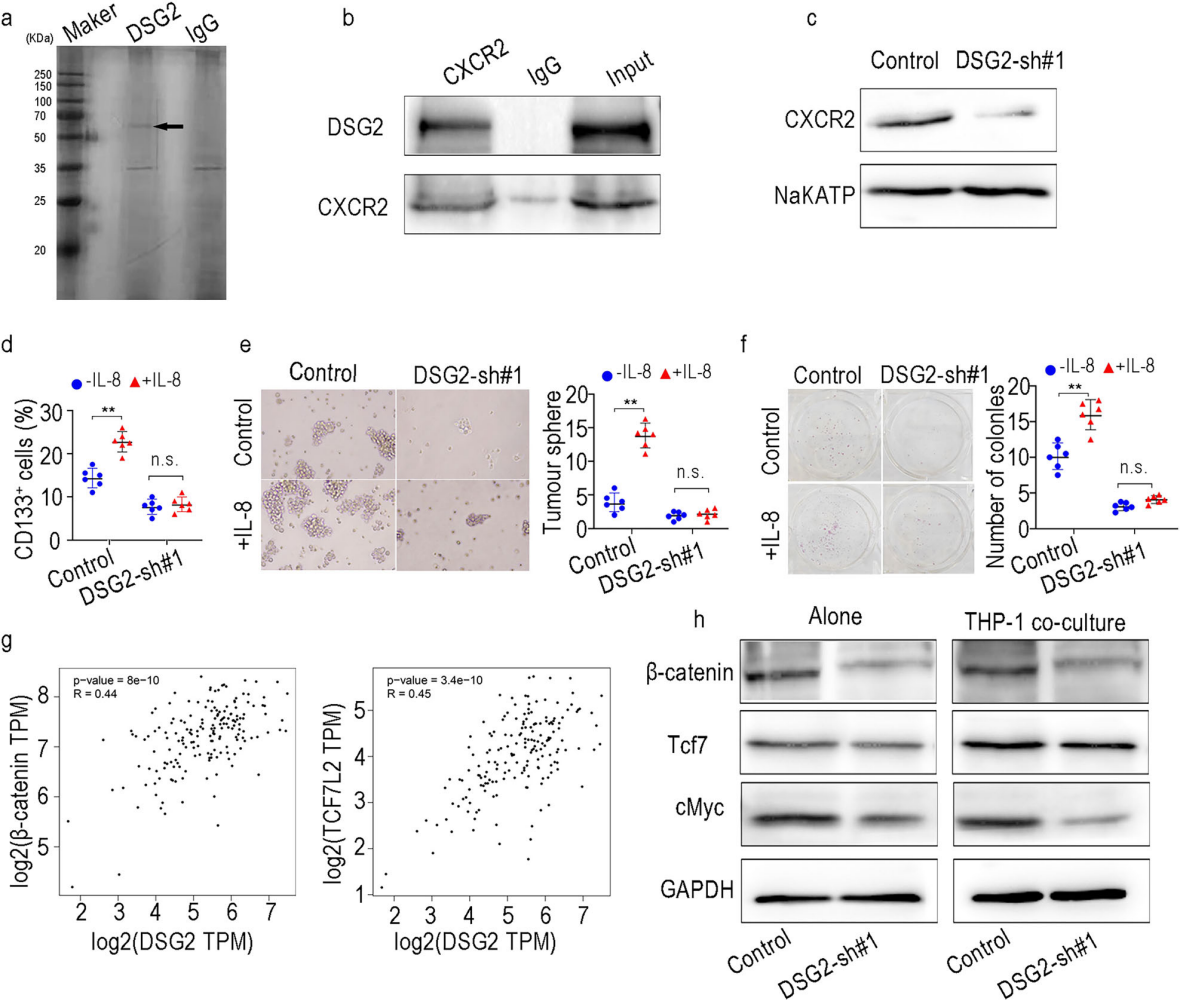
DSG2 promotes macrophage-mediated PCSC stemness by directly binding to CXCR2. **a** A pull-down assay was performed to identify candidates for interaction with DSG2. **b** Co-IP was performed to detect the interaction between DSG2 and CXCR2. **c** The expression levels of CXCR2 in the plasma membrane fraction. **d** The percentages of CD133^+^ cell populations were detected via flow cytometry. **e**, **f** Tumoursphere and colony formation assays in the IL-8 group and control group. **g** The relationships between DSG2 expression and β-catenin and Tcf7 expression were determined via GEPIA. **h** Western blot analyses were performed to assess Wnt/β-catenin signalling pathway activity. The data are presented as the mean ± SD from three independent experiments. *P < 0.05, **P < 0.01.

Pull-down assays were performed with cell membrane lysates to identify candidates for interaction with DSG2 (Fig. 6a). We analysed the mass spectrometry (MS) results and found that CXCR2 was a top-ranked protein. Co-IP confirmed that DSG2 interacts with CXCR2 in PDAC cells (Fig. 6b, c). Given the important role of the IL-8/CXCR2 axis in PCSC stemness [27], we confirmed whether the IL-8/CXCR2 axis is involved in the cross-talk between PCSCs and macrophages regulated by DSG2. Tumoursphere formation and colony formation assays revealed that DSG2 knockdown notably inhibited the increase in PCSC stemness induced by IL-8 (Fig. 6d–f). Previous studies have suggested that the Wnt/β-catenin signalling pathway plays a critical role in the process of PCSC self-renewal [28]. Therefore, we performed a coexpression analysis and found that DSG2 was closely associated with the Wnt/β-catenin signalling pathway (Fig. 6g). The WB results also confirmed that DSG2 knockdown significantly inhibited the expression of β-catenin, Tcf7 and c-Myc (Fig. 6h). These comprehensive investigations robustly establish the indispensable role of the Wnt/β-catenin signalling pathway in driving the DSG2-mediated processes of PCSC stemness maintenance.

### DSG2 enhances the resistance of PDAC cells to gemcitabine

**Fig. 7:**
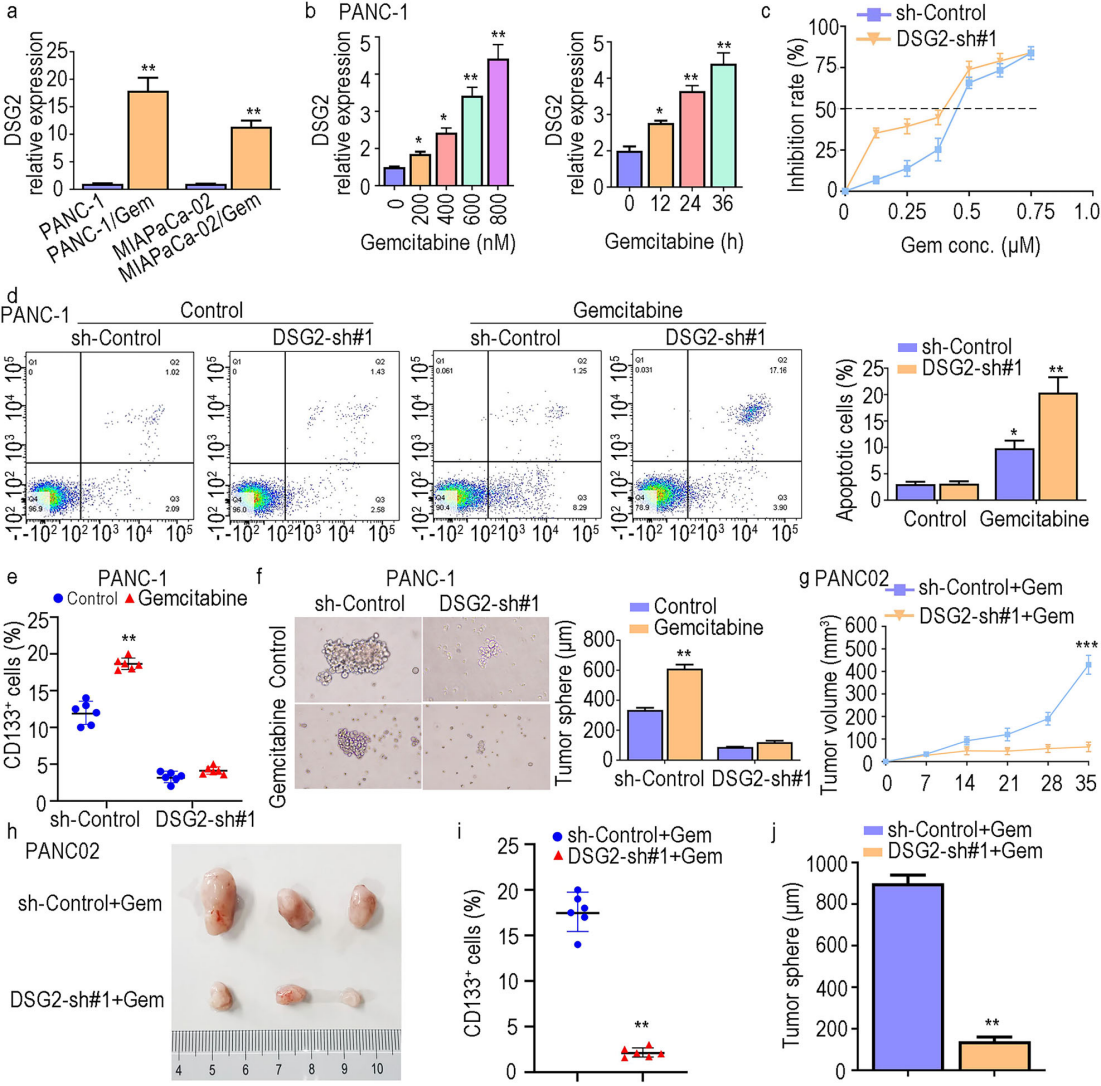
DSG2 promotes the resistance of PDAC cells to gemcitabine. **a** DSG2 expression levels in the gemcitabine-resistant group. **b** Differential expression of DSG2 in PANC-1 cells after treatment with various concentrations of gemcitabine. **c** Cell viability after treatment with different concentrations of gemcitabine. **d** After treatment with gemcitabine, cell apoptosis was detected via flow cytometry. **e** After treatment with gemcitabine, CD133^+^ cells were detected via flow cytometry. **f** Tumoursphere formation assay following treatment with gemcitabine in different groups. **g**, **h** The volume of the tumour xenografts in the different groups. **i** The percentage of CD133^+^ cells in the tumour xenografts. **j** Tumoursphere formation ability of cells in tumour xenografts. The data are presented as the mean ± SD from three independent experiments. *P < 0.05, **P < 0.01, ***P < 0.001.

CSCs are a major reason for resistance to gemcitabine therapy [29, 30]. In the final phase of this study, we focused on assessing the role of DSG2 in gemcitabine resistance. We found that DSG2 expression was significantly increased in gemcitabine-resistant cells (Fig. 7a, b). Compared with that in the control group, the half-maximal inhibitory concentration (IC50) was notably lower in the DSG2-sh#1 group (Fig. 7c). Notably, silencing DSG2 in PANC-1 cells led to an increase in the percentage of apoptotic cells after gemcitabine treatment (Fig. 7d). Moreover, our results revealed that DSG2 knockdown inhibited the enrichment of PCSCs induced by gemcitabine therapy (Fig. 7e, f). Concurrently, to further confirm the contributions of DSG2 to gemcitabine therapy in vivo, we established a tumour xenograft mouse model with PANC02 cells. The results revealed that the absence of DSG2 significantly inhibited tumour growth and relapse (Fig. 7g, h). DSG2 knockdown not only decreased the enrichment but also inhibited the tumoursphere formation ability of PCSCs (Fig. 7I, j). These findings collectively demonstrate that DSG2 plays an important role in PCSC-mediated gemcitabine resistance.

## Discussion

**Fig. 8:**
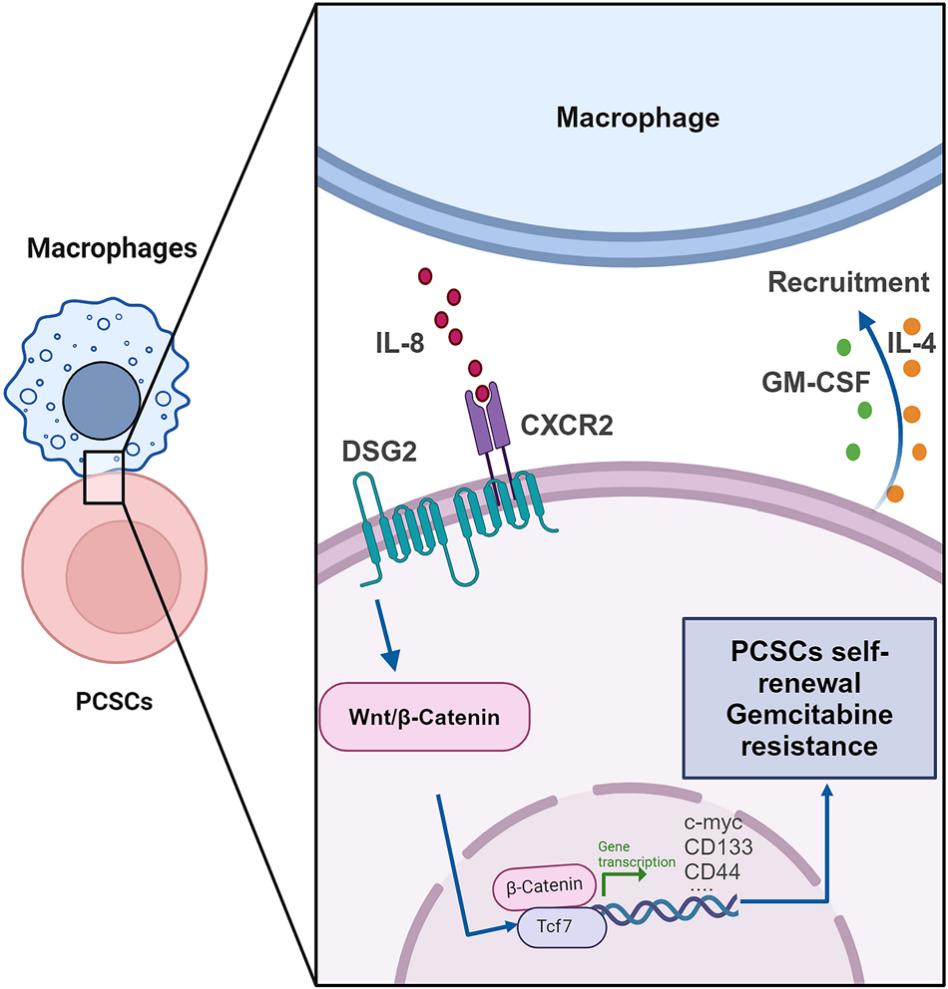
Graphical representation of the working model. DSG2 promotes the secretion of the cytokines IL-4 and GM-CSF, increasing the number of macrophages. The IL-8/CXCR2 axis interacts with DSG2, promoting the stemness and gemcitabine resistance of PCSCs through the Wnt/c-Myc/β-catenin pathway.

PC, a formidable gastrointestinal malignancy, is characterised primarily by the existence of PCSCs [31]. Cross-talk between CSCs and their microenvironment plays a pivotal role in tumour development [32]. By identifying the genes critical for the maintenance of PCSCs and applying multiple publicly available datasets, we were able to identify DSG2 as an oncogenic factor that promotes PC progression. We found that DSG2 mediated the recruitment of macrophages and promoted the formation of the PCSC niche to maintain PCSC stemness (Fig. 8). Additionally, database analysis revealed increased DSG2 expression in PC tissues compared with normal tissues. Patients with high expression of DSG2 have exhibit poor survival outcomes, further supporting its potential as an oncogenic factor in cancer [33]. Notably, DSG2 knockdown increased sensitivity to gemcitabine chemotherapy and inhibited PC cell stemness and tumorigenesis. These findings suggest that abnormal DSG2 expression may be pivotal in the initiation and progression of PC, thus impacting tumour prognosis.

To explore its regulatory mechanisms in the PCSC niche, we first assessed the expression of cytokines, which play critical roles in macrophage recruitment [34]. We found that downregulation of DSG2 resulted in substantial inhibition of IL-4 and GM-CSF, suggesting that DSG2 facilitates the infiltration of macrophages by promoting IL-4 and GM-CSF expression in the PCSC niche. A previous study utilised a pull-down assay and co-IP to confirm the binding partnership between proteins [35]. Our results indicate that DSG2 directly interacts with CXCR2, a key G protein-coupled receptor (GPCR) and the ligand IL-8 [36]. Therefore, CXCR2 was first named interleukin-8 receptor B [37]. CXCR2 has significant protumour functions in a variety of tumours, including non-small cell lung cancer and PC [38, 39]. Notably, recent studies have shown that CXCR2 is also important for the recruitment of macrophages to the tumour niche [40]. Our results revealed that DSG2 knockdown significantly inhibited the stemness of PCSCs treated with IL-8 or cocultured with macrophages, indicating that DSG2 promotes the IL-8/CXCR2 axis.

According to existing research, cytokines and chemokines, especially those involved in the IL8/CXCR2 axis, are vital factors that promote CSC stemness and chemotherapy resistance by activating downstream pathways, including the Wnt/β-catenin pathway [41]. Similarly, the Wnt/β-catenin pathway also plays an important role in DSG2-mediated regulation of CSCs [42]. In line with these findings, our results demonstrate that Wnt/β-catenin expression is significantly inhibited in the DSG2-downregulation group, emphasising the oncogenic role of Wnt/β-catenin in PCSCs. These findings strongly indicate the involvement of DSG2 in mediating the malignant phenotypes of PCSCs through the Wnt/β-catenin pathway. Therefore, DSG2 mediates the expression of IL-4 and GM-CSF and promotes the recruitment of macrophages to facilitate formation of the PCSC niche. In summary, the enrichment of macrophages activates the IL-8/CXCR2 axis, and increased DSG2 expression leads to the maintenance of PCSC stemness through the Wnt/β-catenin pathway (Fig. 8).

## Conclusions

In summary, our study revealed a significant difference in DSG2 expression levels between PCSCs and non-CSCs. This disparity highlights that downregulating DSG2 expression may suppress PC cell metastasis. Furthermore, our findings elucidated a novel mechanism involving the IL-8/CXCR2-DSG2-Wnt/β-catenin axis, which plays a crucial role in the formation of the PCSC niche. Consequently, targeting DSG2 could offer a promising avenue for more effective and targeted therapies in the treatment of PC.

## Supplementary information

Supplementary Information

raw data of WB

## Data Availability

All data produced or analysed in this study are contained in this published article and its supplementary information files. The datasets used and/or analysed in this research are accessible by the corresponding authors upon reasonable request.
