# Supplementary material for: DSG2 promotes pancreatic cancer stem cell maintenance via support of tumour and macrophage cellular cross-talk
Source: Cell Death Dis. 2025 Jul 4;16(1):492. doi: 10.1038/s41419-025-07833-4 (PMC12227620; doi:10.1038/s41419-025-07833-4)
Supplement: Supplementary file 1 — Supplementary Information [file 41419_2025_7833_MOESM1_ESM.docx]

**Supplementary Information**

**DSG2 promotes pancreatic cancer stem cell maintenance via support of tumour and macrophage cellular cross-talk**

Faming Wang^a-b^, Tao Sun^c^, Ning Wang^a-b^, Wei Wei^a-b^, Ying Mei^a^, Qiang Yan^d-g*^

List of Supplementary Material

**1）Supplementary Figures**

Supplementary Figure 1, related to Figure 1

Supplementary Figure 2, related to Figure 2

Supplementary Figure 3, related to Figures 3

Supplementary Figure 4, related to Figures 5

**2）Supplementary Tables**

Table S1. Primer sequences for qRT-PCR

Table S2. Antibodies used in all related experiments.

1. **Supplemental Figures**

Supplementary Figure 1, related to Figure 1


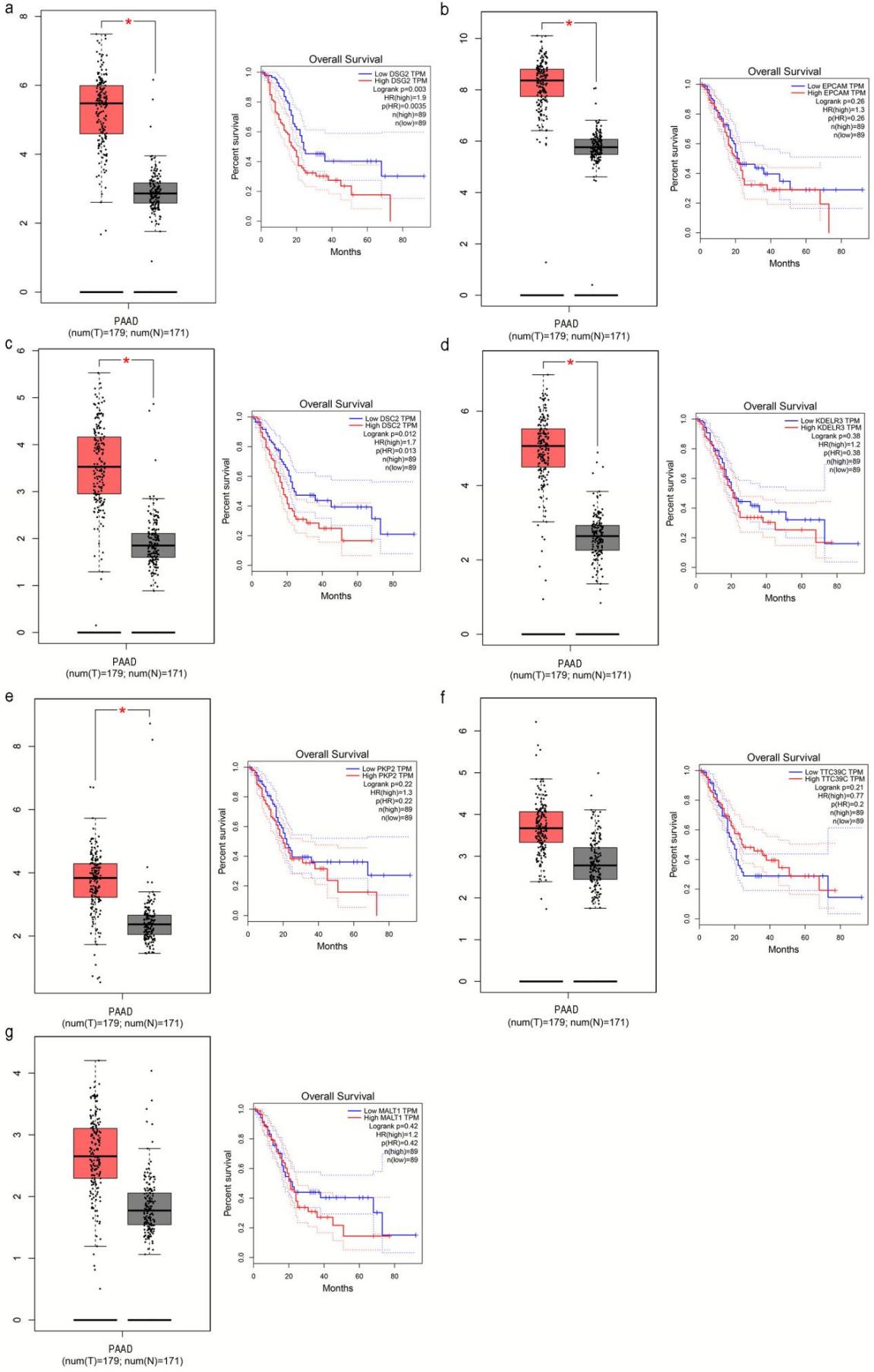


Figure S1. Impact of different genes expression on survival outcomes in PAAD patients. (a) The expression levels of DSG2 and the Impact of its high expression on survival probability in PAAD patients. (b) The expression levels of EPCAM and the Impact of its high expression on survival probability in PAAD patients. (c) The expression levels of DSC2 and the Impact of its high expression on survival probability in PAAD patients. (d) The expression levels of KDELR3 and the Impact of its high expression on survival probability in PAAD patients. (e) The expression levels of PKP2 and the Impact of its high expression on survival probability in PAAD patients. (f) The expression levels of TTC39C and the Impact of its high expression on survival probability in PAAD patients. (g) The expression levels of MALT1 and the Impact of its high expression on survival probability in PAAD patients.

Supplementary Figure 2, related to Figure 2


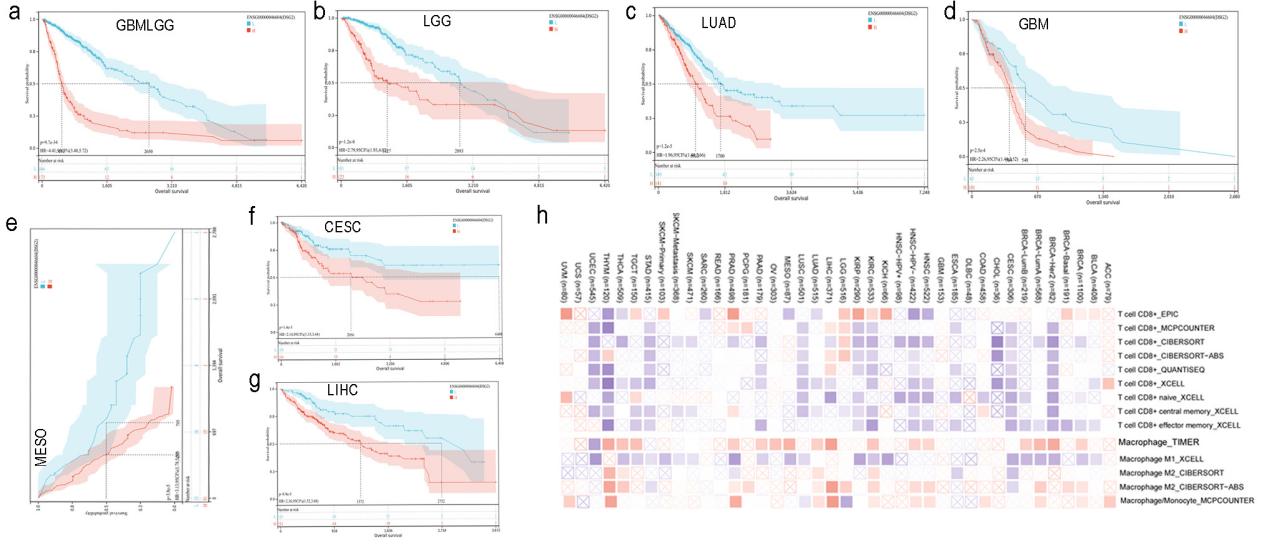


Figure S2.(a) Impact of high DSG2 expression on survival probability in patients with GBM and LGG. (b) Impact of high DSG2 expression on the survival probability of patients with LGG. (c) Impact of high DSG2 expression on the survival probability of patients with LUAD. (d) Impact of high DSG2 expression on the survival probability of patients with GBM. (e) Impact of high DSG2 expression on the survival probability of patients with MESO. (f) Impact of high DSG2 expression on the survival probability of patients with CESE. (g) Impact of high DSG2 expression on the survival probability of patients with LIHC. (h) Correlation between immune cell infiltration and DSG2 expression in tumors obtained through TIMER2.0 online tool. TIMER2.0 integrates six advanced immune deconvolution algorithms (including TIMER, CIBERSORT, quanTIseq, xCell, MCP-counter, and EPIC) to estimate the levels of immune cell infiltration in tumor samples. Macrophages outperform other immune cells. Abbreviations: DSG2, Desmoglein-2; GBMLGG, Brain lower grade glioma; LUAD, Lung adenocarcinoma; LIHC, Liver hepatocellular carcinoma; CESC, Cervical squamous cell carcinoma and endocervical adenocarcinoma; MESO, Mesothelioma; GBM, Glioblastoma multiforme

Supplementary Figure 3, related to Figure 3


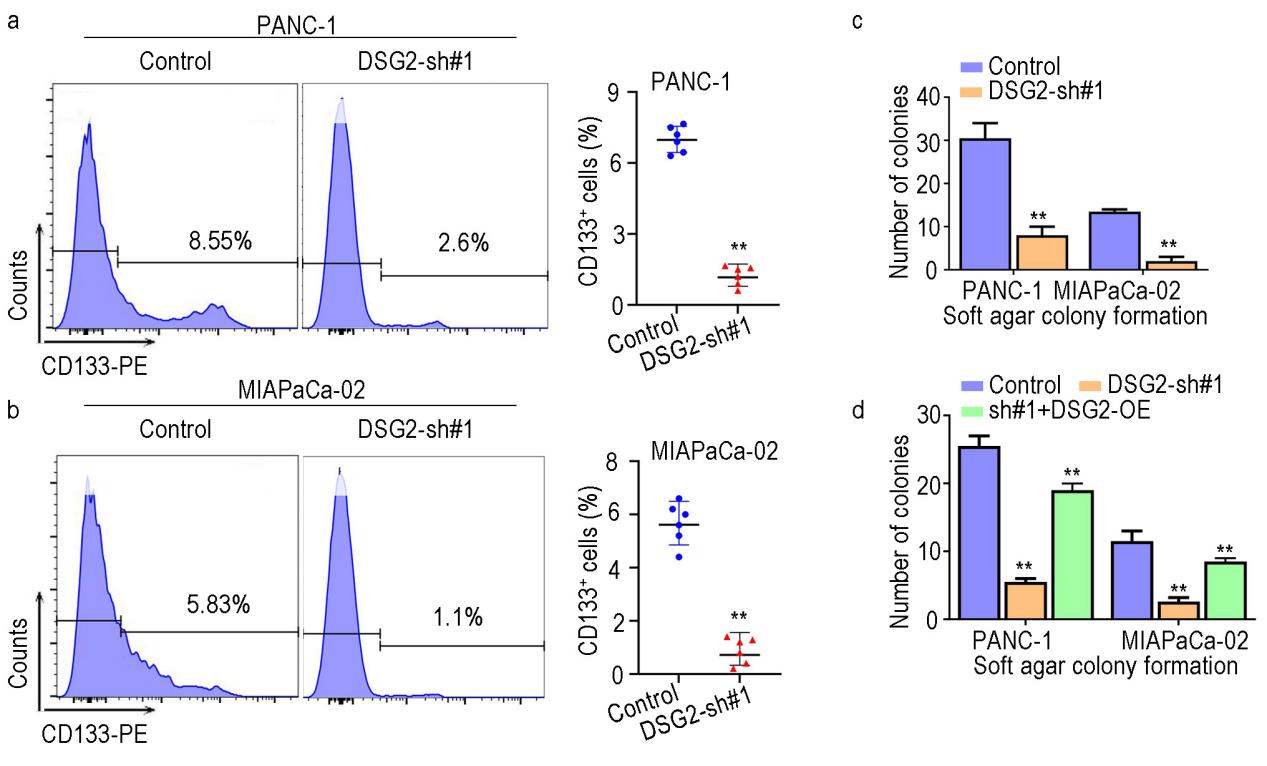


Figure S3. DSG2 knockdown inhibits the stemness of PCSCs in vitro. (a-b) CD133+ population of PANC-1 and MIAPaCa-02 cells were assessed through flow cytometry assays. (c-d) Soft agar colony formation assays were carried out to validate the role of DSG2 in the proliferation of PANC-1 and MIAPaCa-02 cells. The data are presented as mean ± SD (standard deviation) from three independent experiments. *P < 0.05, **P < 0.01.

Supplementary Figure 4, related to Figures 5


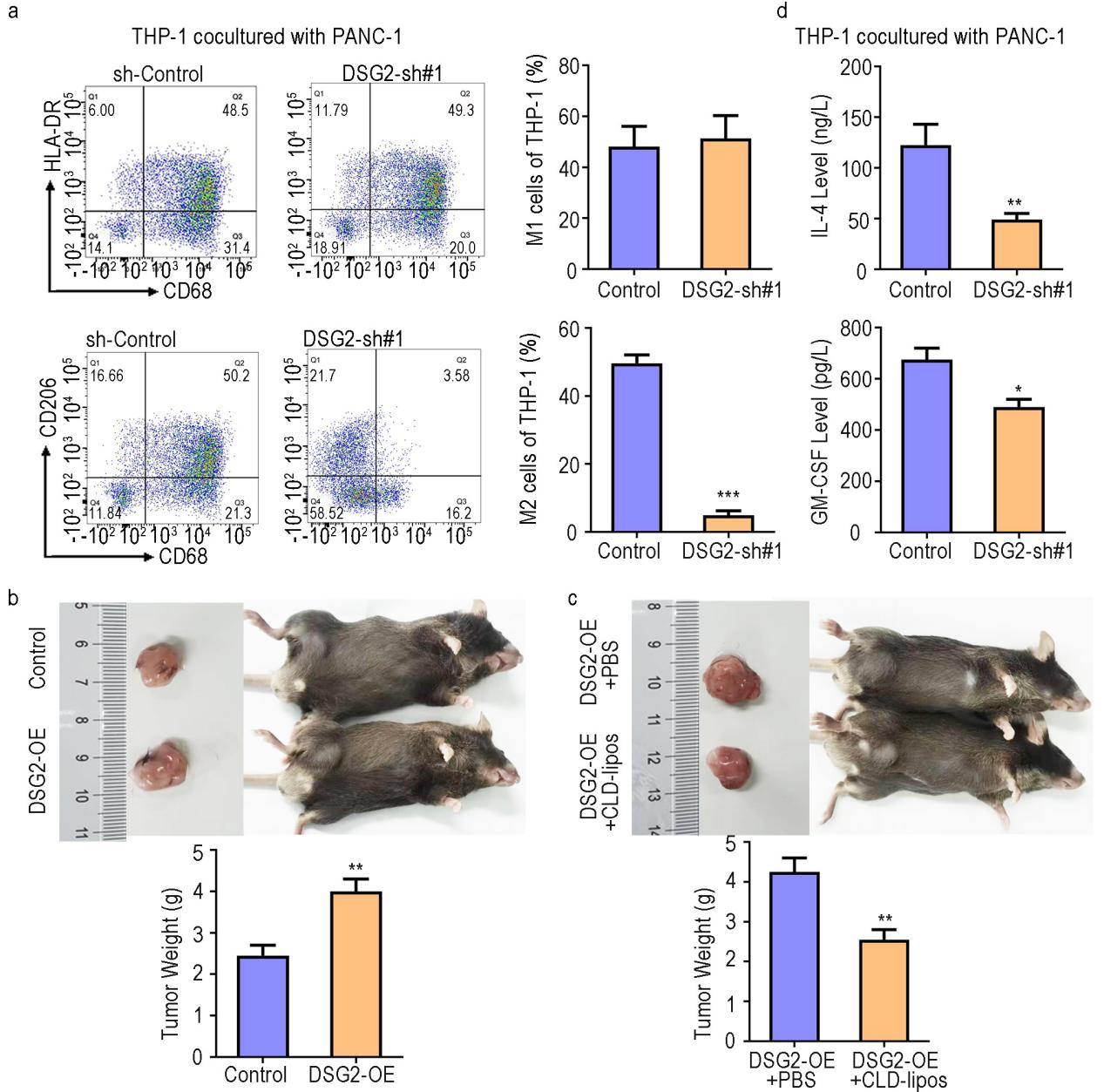


Figure S4. The effect of DSG2 knockdown on the number of M2 macrophages and related factors. (a) Flow cytometry analysis revealed that the number of M2 macrophages in the DSG2 knockdown group was significantly lower than that in the control group, indicating that the absence of DSG2 significantly inhibits the differentiation or survival of M2 macrophages. (b-c) The volume of the tumour xenografts in the different groups. (d) ELISA experiments was used to detected the levels of cytokines in the cell co-culture supernatant. Compared with the control group, the levels of cytokines (IL-4 and GM-CSF) in the DSG2 knockdown group were significantly reduced. The data are presented as mean ± SD (standard deviation) from three independent experiments. *P < 0.05, **P < 0.01.

**2）Supplementary Tables**

**Table S1**. Primer sequences for qRT-PCR

| Gene name | Forward primer (5'→3') | Reverse primer (5'→3') |
| --- | --- | --- |
| DSG2-shRNA#1 | CCGGAGATGATTTGGGACTTAAATTCTCGAGAATTTAAGTCCCAAATCATCTTTTTTG | AATTCAAAAAAGATGATTTGGGACTTAAATTCTCGAGAATTTAAGTCCCAAATCATCT |
| DSG2 | ACGTAGAAGTTACGCGCATAAA | GGGTCACAATTCCTTCGTTAGTT |
| GAPDH | CTGGGCTACACTGAGCACC | AAGTGGTCGTTGAGGGCAATG |
| CD133 | AGTCGGAAACTGGCAGATAGC | GGTAGTGTTGTACTGGGCCAAT |
| CD44 | CTGCCGCTTTGCAGGTGTA | CATTGTGGGCAAGGTGCTATT |
| EpCAM | AATCGTCAATGCCAGTGTACTT | TCTCATCGCAGTCAGGATCATAA |
| CXCL1 | AACCGAAGTCATAGCCACA | TCCTAAGCGATGCTCAAA |
| CXCL2 | ACAGAGCCCGGGCCACAGGCAGCTC | GAGCTGCCTGTGGCCCGGGCTCTGT |
| CXCL3 | CGCCCAAACCGAAGTCATAG | GCTCCCCTTGTTCAGTATCTTTT |
| CXCL5 | AGCTGCGTTGCGTTTGTTTAC | TGGCGAACACTTGCAGATTAC |
| IL-4 | CCAACTGCTTCCCCCTCTG | TCTGTTACGGTCAACTCGGTG |
| GM-CSF | CTCCTTTGGCCTATTCTACAAGC | TGAACAGAGACGATGTATTGGC |
| IL-6 | ACTCACCTCTTCAGAACGAATTG | CCATCTTTGGAAGGTTCAGGTTG |
| IL-10 | TCAAGGCGCATGTGAACTCC | GATGTCAAACTCACTCATGGCT |

**Table S2**. Antibodies used in all related experiments.

| Antibody name | Corporation name | Catalog number | Dilution ratio |
| --- | --- | --- | --- |
| DSG2 | Proteintech | 68515-1-Ig | 1:1000 |
| Ki-67 | Abcam | ab15580 | 1:3000 |
| IL-4 | Abcam | ab34277 | 1:3000 |
| GM-CSF | Abcam | ab316862 | 1:3000 |
| Tcf7 | Proteintech | 84089-4-RR | 1:800 |
| C-Myc | Proteintech | 80845-1-RR | 1:1000 |
| β-Catenin | Abcam | ab246504 | 1:3000 |
| GAPDH | Proteintech | 60004-1-Ig | 1:5000 |
| Goat anti-mouse IgG | Abbkine | A21010 | 1:5000 |
| Goat anti-rabbit IgG | Abbkine | A21020 | 1:5000 |
