# Supplementary figures and images for: DSG2 promotes pancreatic cancer stem cell maintenance via support of tumour and macrophage cellular cross-talk
Source: Cell Death Dis. 2025 Jul 4;16(1):492. doi: 10.1038/s41419-025-07833-4 (PMC12227620; doi:10.1038/s41419-025-07833-4)

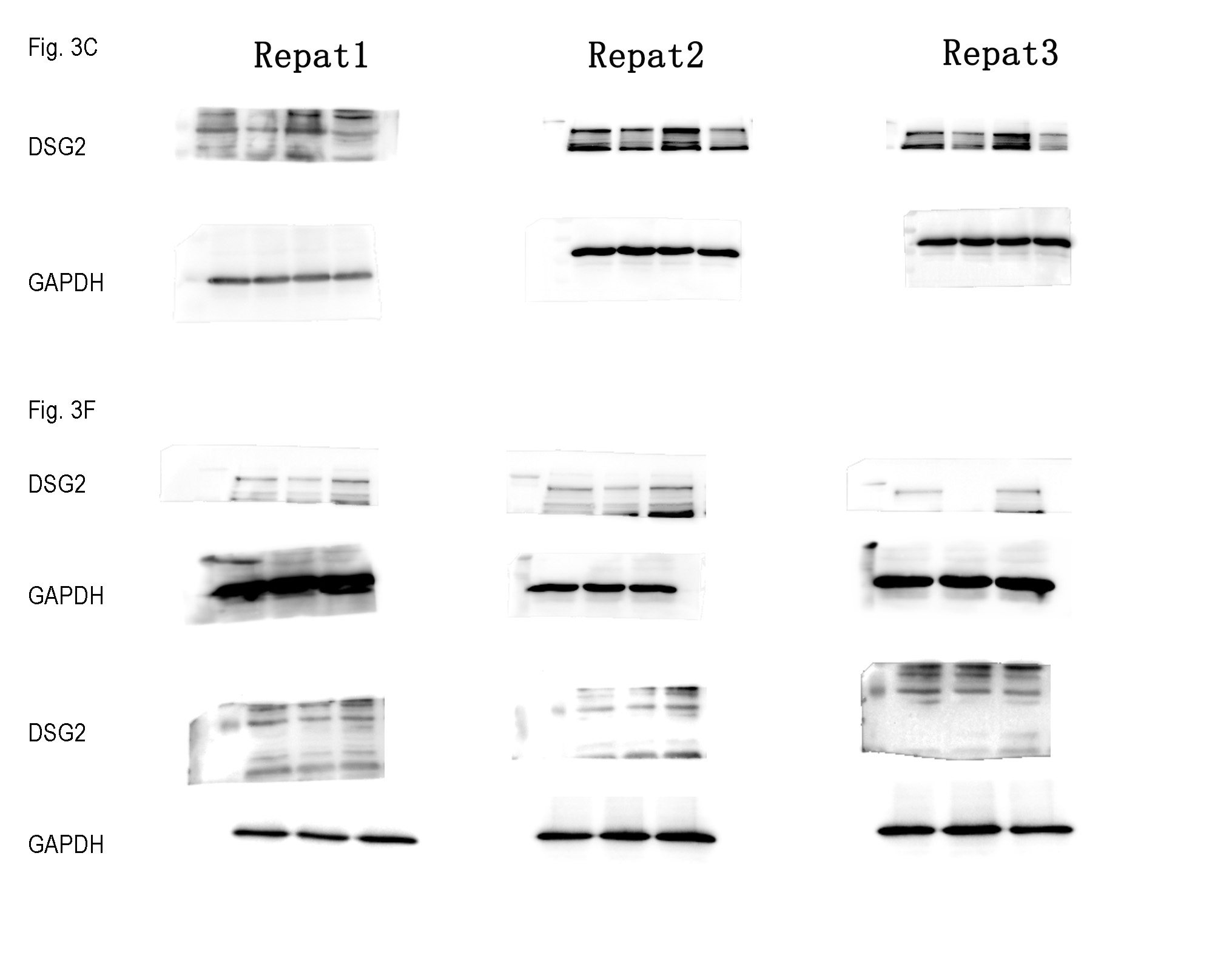


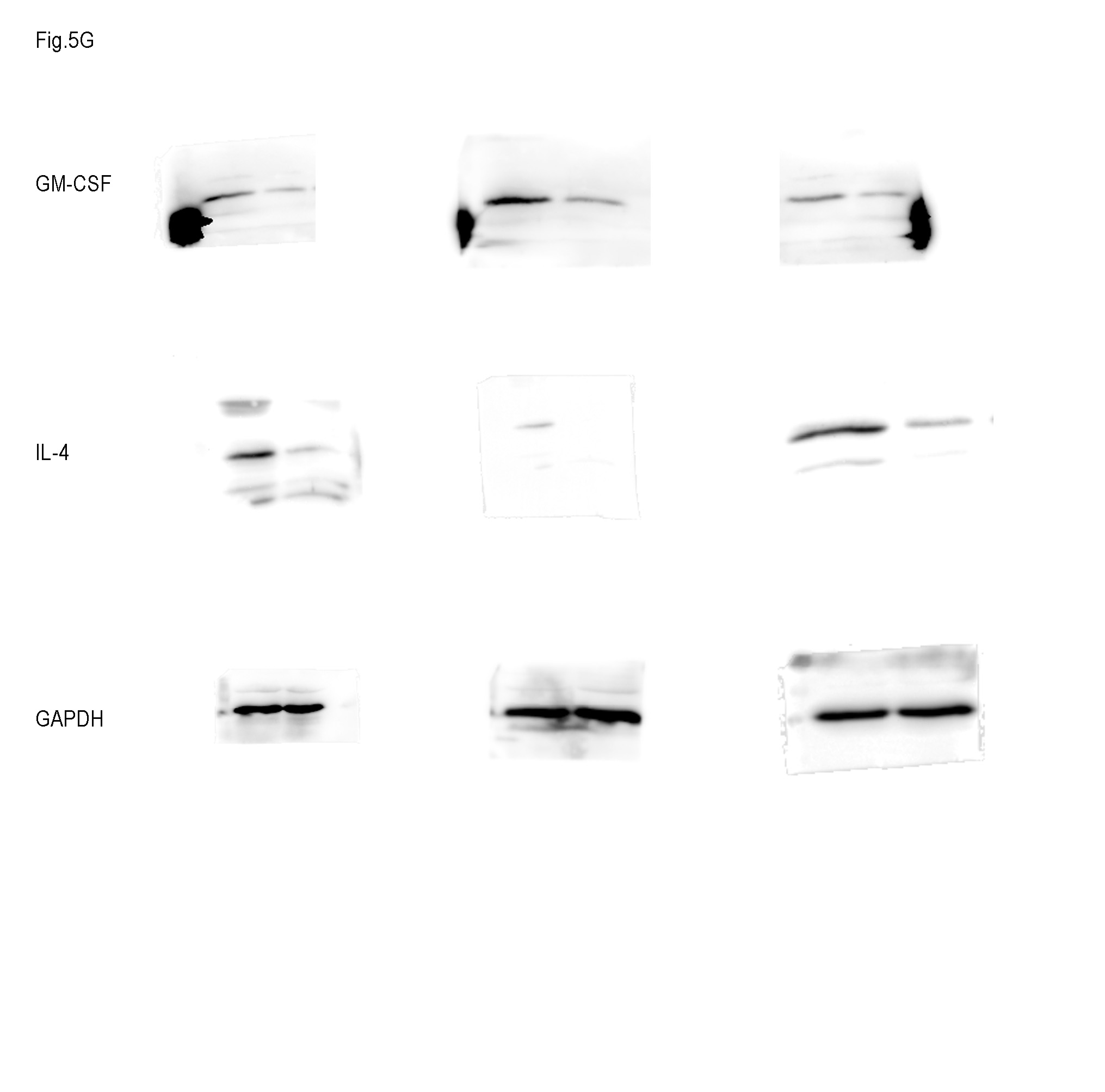


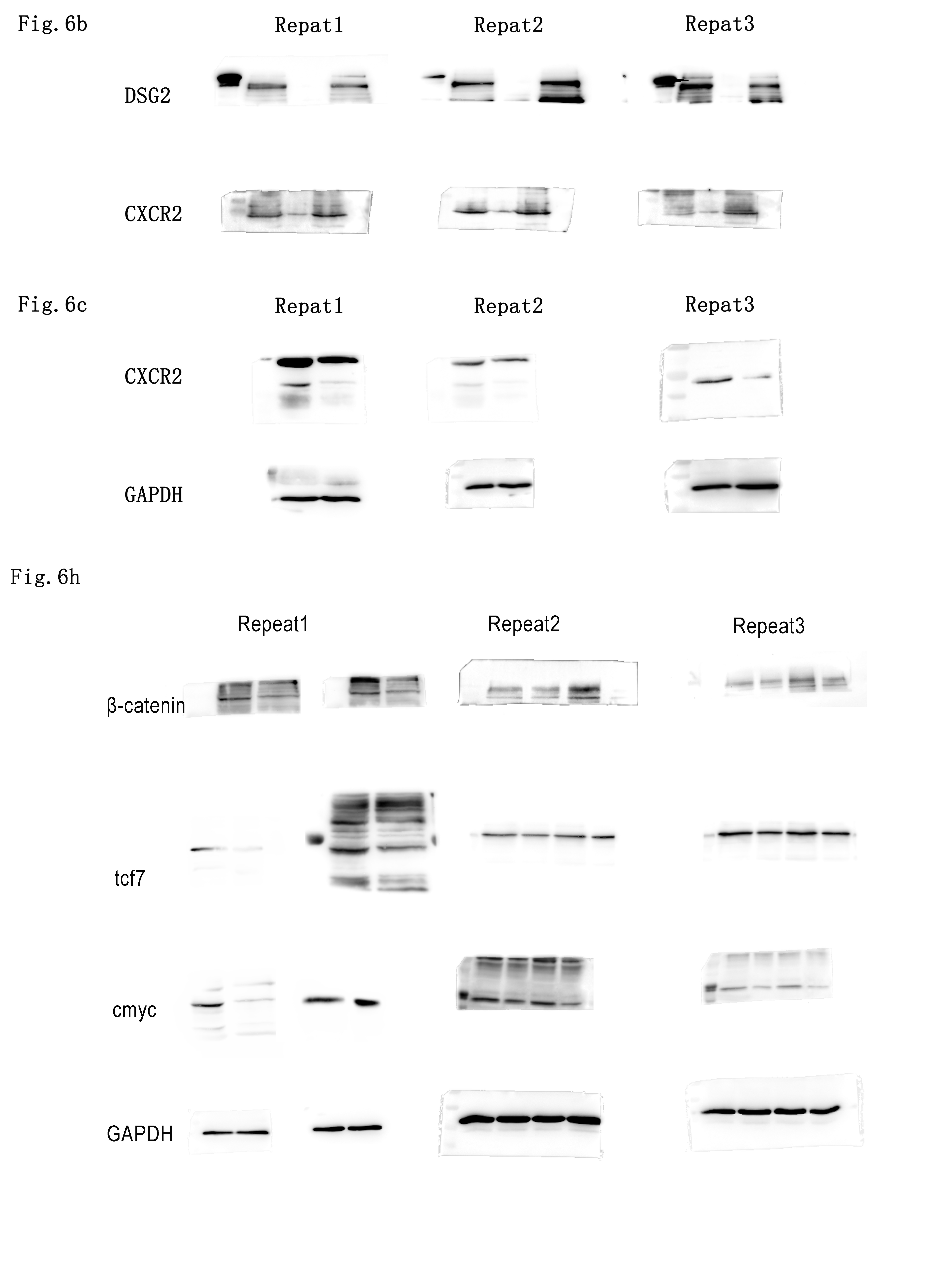

Supplement: Supplementary file 2 — raw data of WB [file 41419_2025_7833_MOESM2_ESM.doc]
